# Interplay between the heterotrimeric G-protein subunits G_αq _and G_αi2 _sets the threshold for chemotaxis and TCR activation

**DOI:** 10.1186/1471-2172-10-27

**Published:** 2009-05-08

**Authors:** Jacob Ngai, Marit Inngjerdingen, Torunn Berge, Kjetil Taskén

**Affiliations:** 1 The Biotechnology Centre of Oslo, University of Oslo, N-0317 Oslo, Norway; 2 Centre for Molecular Medicine, Nordic EMBL Partnership, University of Oslo, N-0317 Oslo, Norway; 3 Institute of Immunology (IMMI), Rikshospitalet-Radiumhospitalet Medical Centre, University of Oslo, N-0027 Oslo, Norway

## Abstract

**Background:**

TCR and CXCR4-mediated signaling appears to be reciprocally regulated pathways. TCR activation dampens the chemotactic response towards the CXCR4 ligand CXCL12, while T cells exposed to CXCL12 are less prone to subsequent TCR-activation. The heterotrimeric G proteins G_αq _and G_αi2 _have been implicated in CXCR4-signaling and we have recently also reported the possible involvement of G_αq _in TCR-dependent activation of Lck (Ngai et al., Eur. J. Immunol., 2008, 38: 32083218). Here we examined the role of G_αq _in migration and TCR activation.

**Results:**

Pre-treatment of T cells with CXCL12 led to significantly reduced Lck Y394 phosphorylation upon TCR triggering indicating heterologous desensitization. We show that knockdown of G_αq _significantly enhanced basal migration in T cells and reduced CXCL12-induced SHP-1 phosphorylation whereas G_αi2 _knockdown inhibited CXCL12-induced migration.

**Conclusion:**

Our data suggest that G_αi2 _confers migration signals in the presence of CXCL12 whereas G_αq _exerts a tonic inhibition on both basal and stimulated migrational responses. This is compatible with the notion that the level of G_αq _activation contributes to determining the commitment of the T cell either to migration or activation through the TCR.

## Background

Chemokines dictate the migration and positioning of leukocytes at steady-state and in inflammation [1]. The chemokine CXCL12, also known as stromal cell-derived factor-1α (SDF-1α), is a member of the CXC chemokine subfamily and serves as the only known ligand for the chemokine receptor CXCR4 expressed on most leukocytes [1-3]. Mice lacking CXCL12 or CXCR4 die perinatally of cardiovascular, neurologic, vascular, and hematopoietic defects [4,5]

The CXCR4 receptor is a G-protein coupled receptor (GPCR) and couples to heterotrimeric G-proteins of both the G_αi _and G_αq_-families [6-8]. Members of the G_αi_-family of G-proteins are pertussis toxin sensitive and pre-treatment of cells with pertussis toxin severely attenuates chemotaxis in response to chemokines. G_αi_-proteins are therefore thought to be essential for chemokine-mediated migration. Of the G_αi_-family, G_αi2 _and G_αi3 _are strongly expressed by lymphocytes [9]. G_αi3_^-/- ^mice are without phenotype [10] whereas G_αi2_^-/- ^mice display dysfunctional T cell functions as well as defective chemotaxis in response to chemokines [6]. In contrast, G_αq _has been shown to be required for chemotaxis of dendritic cells and granulocytes, but not for T cells [7].

Stimulation of the T cell receptor (TCR), the B cell receptor, or the Ly49D activating receptor all reduce lymphocyte migration toward CXCL12 [11-14] suggesting that cells dedicated to proliferation/differentiation are less sensitive towards chemokines. The opposite is also true, as cells exposed to CXCL12 show less phosphorylation of ZAP-70 and LAT following subsequent TCR activation indicating reciprocal regulation between CXCR4 and the TCR [13]. In addition, recruitment of chemokine receptors into the immunological synapse has been shown to stabilize T cell-APC interaction through activation of a G_αq_-dependent pathway. This renders the cell less responsive to chemotactic gradients and gives higher T cell proliferative responses and cytokine production [8]. Recently, the TCR and CXCR4 were shown to closely associate upon CXCL12-stimulation [15], and it was suggested that CXCR4 signaling occurs through the immunoreceptor tyrosine-based activation motif (ITAM)-domain present in the CD3ζ chain. Collectively, these results point to a direct crosstalk between the signaling machineries utilized by the TCR and CXCR4.

We have recently shown that G_αq _knockdown in T cells inhibits the activation of the Src family kinase Lck [16]. The effect of G_αq _on Lck activation might be conferred by a direct interaction and/or by affecting the threshold for TCR-triggering. We demonstrate here that knockdown of G_αq _significantly enhances basal T cell migration while inhibiting TCR-induced Lck activation. Pre-treating cells with CXCL12 prior to stimulation with anti-CD3 leads to a similar inhibition of Lck activation indicating that cells directed to migrate are less responsive to TCR triggering. The signals conveyed by G_αq _appear to be mediated through a SHP-1 pathway. Our data suggest that optimal TCR activation requires signaling through G_αq_, and that removal of G_αq _locks the cell in migration modus making the cell less responsive to TCR signaling

## Methods

### Antibodies and reagents

Human recombinant CXCL12 was obtained from Invitrogen (cat. no. PHC1344) and Peprotech (Rocky Hill, NJ). Antibody to G_αi2 _was from Calbiochem (cat. no. 371727). Antibodies against SH-PTP1, G_αq_, Lck, Protein A/G Plus Agarose beads and SH-PTP1 conjugated agarose beads were purchased from Santa Cruz (cat. no. sc-287, cat. no. sc-393, cat. no. sc-433, cat. no. sc-2003, cat. no. sc-287 AC). Antibodies against phospho-p44/42 MAPK (Thr202/Tyr204) and Src pY416 (detecting also Lck pY394) were obtained from Cell Signaling (cat. no. 9106 and cat. no. 2101). Anti-phosphotyrosine mAb (4G10) was purchased from Upstate (cat. no. 05-777). Peroxidase-conjugated secondary antibodies and Affinipure F(ab')_2 _fragment Goat Anti-Mouse IgG were obtained from Jackson ImmunoResearch Laboratories (cat. no. 111-035-144, cat. no. 115-035-146, cat. no. 115-006-072). Anti-CXCR4-PE was obtained from BD Pharmingen (cat. no. 555974).

### siRNA design

21-nt siRNA duplexes targeting human G_αq _mRNA (NM_002072) (GNAQ-1103; 5'-GGAGUACAAUCUGGUCUAAUU-3'; 5'-UUAGACCAGAUUGUACUCCUU-3') and a triple G/C switch control (GNAQ-1103M3; 5'-GCAGUAGAAUCUGCUCUAAUU-3'; 5'-UUACACCACAUUGUAGUCCUU-3'); G_αi2 _mRNA (NM_002070.1) (GNAI-1050; 5'-GGACCUGAAUAAGCGCAAAGA-3'; 5'-UUUGCGCUUAUUCAGGUCCUC-3') were designed and synthesized in-house. The oligos are named according to the position of the 5' nucleotide of the sense strand relative to the reference sequences. For a detailed characterization of the effect of these siRNAs, see Ngai et al. [16].

### Cell culture and transfections

The Jurkat TAg T cell line stably transfected with SV40 large T antigen was kept in logarithmic growth in RPMI-1640 (Invitrogen) supplemented with 10% FCS, 1% Penicillin-Streptomycin, 1% Non-essential amino acids and 1% Sodium Pyruvate. 20 × 10^6 ^cells were washed and resuspended in 400 μl OptiMEM (Invitrogen), mixed with cDNA or siRNA and electroporated in 0.4 cm electroporation cuvettes (Bio-Rad) at 250 V/cm and 975 μF. Cells were harvested 48 hours post transfection.

### Cell stimulation and lysis

Cells were resuspended in RPMI 1640 to 50 × 10^6^/ml and pre-incubated at 37°C for 5 minutes. Cells were then stimulated with 100 ng/ml CXCL12 or 1.5 μg/ml OKT3 for the indicated time points and the reaction was subsequently stopped by lysis in ice cold lysis buffer (50 mM HEPES, 100 mM NaCl, 5 mM EDTA, 50 mM NaF, 10 mM Na-pyrophosphate, 1 mM Na_3_VO_4_, 1 mM PMSF, 1.0% Triton X-100). Where indicated, cells were pre-treated with 100 ng/ml CXCL12 or 10 μM PP2 for 30 minutes at 37°C prior to stimulation. Samples were spun down at 13 000 rpm for 10 min and the supernatant boiled with SDS-buffer, subjected to SDS-PAGE and transfered to PVDF filters. Filters were subjected to immunoblotting with the indicated antibody over night at 4°C, washed 3 × 10 minutes with TBS-T, incubated 45 minutes with secondary antibody at RT, washed 3 × 10 minutes with TBS-T before incubation with SuperSignal West Pico Chemiluminescent Substrate for 5 minutes.

### Intracellular Ca^2+ ^measurement

Cells were resuspended to 10^7 ^cells/ml in PBS w/1% BSA and incubated with 4 μg/ml Fluo-4 AM and 10 μg/ml Fura Red AM for 30 minutes at 30°C. Cells were spun down and resuspended to 3 × 10^7 ^cells/ml in PBS w/1% BSA. Cells were stimulated with 100 ng/ml CXCL12 and Ca^2+ ^levels were measured by flow cytometry.

### Migration assay

Migration assays were performed in 24-well plates with 5.0 μm pore-sized polycarbonate membrane inserts (Costar Transwell, Cat. no. CLS-3421). Cells were washed in RPMI-1640 supplemented with 2% FCS and 25 mM Hepes (chemotaxis medium) and resuspended to 1 × 10^6 ^cells/ml. Six hundred μl chemotaxis medium was added to wells with or without 10 ng/ml CXCL12. Cells (1.5 × 10^5^) were loaded into the transwell inserts and allowed to migrate for 2 hrs at 37°C and 5% CO_2_. Cells that had passed through the filter into the lower chamber were collected and 10 μl of an internal bead control (Bangs Laboratories, Fishers, IN) was added. The cells were counted by flow cytometry (FACS Calibur, Becton Dickinson) and normalized with reference to the internal bead control. Migration index was calculated as the number of cells migrating towards CXCL12 in the test well relative to the number of unmanipulated cells migrating towards medium in the control wells.

### CXCR4 surface expression

Forty-eight hours post-transfection, Jurkat T cells were stimulated with 100 ng/ml CXCL12 for different time points, washed in ice-cold PBS, fixed in 4% paraformaldahyde for 10 minutes at 37°C, washed in PBS, and resuspended in PBS w/1% BSA and anti-CXCR4-PE for 30 min at 4°C. Thereafter the cells were washed in PBS w/1% BSA and subjected to flow cytometric analysis for detection of surface CXCR4 expression.

### Statistical analysis

All data are representative of at least three different experiments. Values are expressed as mean ± S.E.M as indicated in the figures. Statistical analysis was performed with Student's *t*-test.

## Results and Discussion

### CXCL12-pretreatment attenuates TCR-induced Lck activation comparable to the G_αq_-kd-kd phenotype

**Figure 1:**
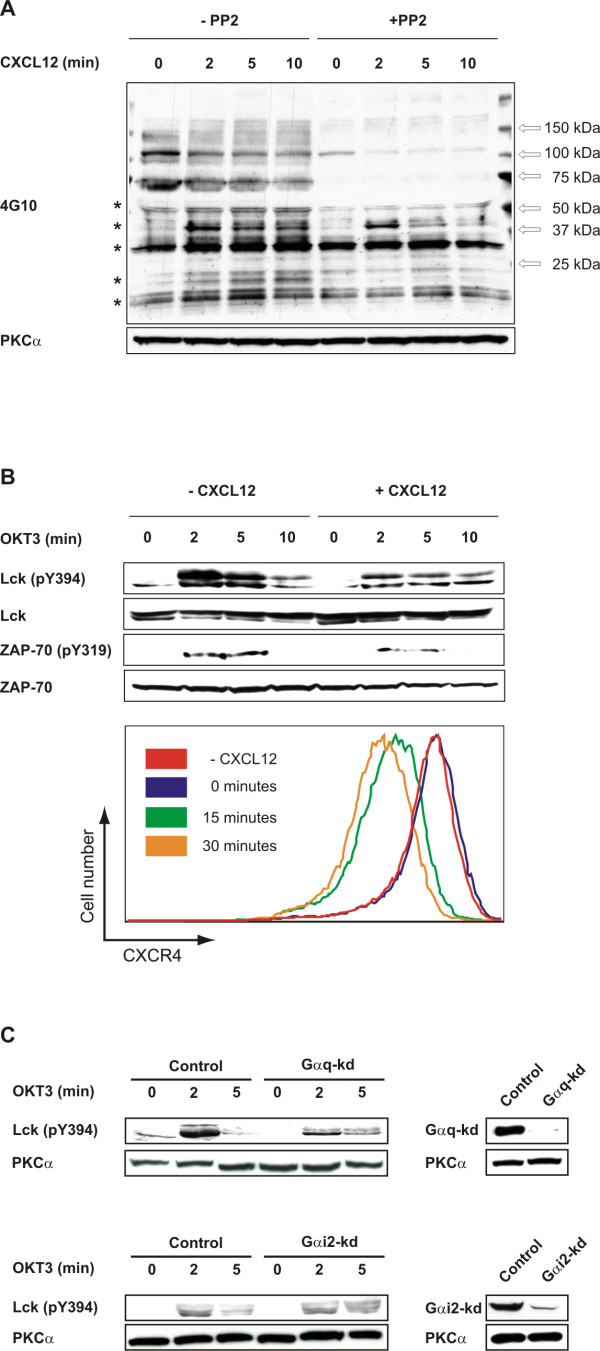
**CXCL12-pretreatment attenuates TCR-induced Lck activation.** (A) Jurkat TAg T cells were incubated with or without 10 &#956M PP2 for 30 minutes at 37°C prior to stimulation with CXCL12 (100 ng/ml) for the indicated time-points and stopped with lysis buffer. Cell lysates were subjected to immunoblotting with the indicated antibodies. (B) Jurkat TAg T cells were incubated with 100 ng/ml CXCL12 for 30 minutes at 37°C prior to stimulation with OKT3 (1.5 μg/ml) for the indicated time-points and stopped with lysis buffer. Cell lysates were subjected to immunoblotting with the indicated antibodies. Cells were also stained with anti-CXCR4 and surface expression of the receptor was analysed by flow cytometry. (C) Jurkat TAg T cells were transfected with G_αq_-specific siRNA (GNAQ1103), G_αi2_-specific siRNA (GNAI-2-1050) or control siRNA (GNAQ1103 M3). 48 hours post-transfection cells were stimulated with OKT3 (1.5 μg/ml) for the indicated time-points and stopped with lysis buffer. Cell lysates were subjected to immunoblotting with the indicated antibodies. All figures are representative of at least 3 independent experiments.

We have previously shown that siRNA-mediated knockdown of the heterotrimeric G protein G_αq _in T cells attenuates TCR-induced Lck activation [16]. While the TCR is not thought to be directly associated with G proteins, the chemokine receptor CXCR4 can couple to both G_αq _and G_αi2_. Furthermore, CXCR4 has been shown to associate with TCR upon CXCL12 activation [15]. Activation of tyrosine kinases by G_α_-subunits has been demonstrated [17-20] and could represent the mechanism by which CXCR4 activates Lck [21]. We show here that the Src family kinase inhibitor, PP2, abrogated CXCL12-induced tyrosine-phosphorylation of several proteins (Figure 1A, marked with *), a further indication that Src family kinases act downstream of CXCR4. However, the changes in tyrosine phosphorylation following CXCL12 treatment are modest compared to those of anti-CD3 treatment (data not shown). Furthermore, pre-treatment of Jurkat T cells with CXCL12 prior to stimulation with anti-CD3 attenuated TCR-induced Lck activation (Figure 1B). Similarly, we also observed reduced ZAP-70 phosphorylation in cells pre-stimulated with CXCL12 (Figure 1B). This is in line with a previous report showing reduced TCR-induced phosphorylation of ZAP-70 and LAT in CXCL12-pretreated cells [13] suggesting that post-activation heterologous desensitization is occurring possibly as a result of G_αq _sequestration. Consistent with this notion surface expression of CXCR4 was also down after 30 minutes incubation with CXCL12. To address the role of G_α_-subunits in controlling the switch between TCR and CXCR4-signaling events, we subjected Jurkat T cells to siRNAs against G_αq _or G_αi2_. We achieved more than 90% knockdown with these siRNAs as analysed by Western blotting (Fig. 1C). As previously demonstrated [16] TCR-induced Lck Y394 autophosphorylation was reduced in G_αq_-knockdown but not G_αi2_-knockdown T cells demonstrating that G_αq _may directly or indirectly activate Lck (Fig 1C).

### G_αq_-kd sets the threshold for chemotaxis

**Figure 2:**
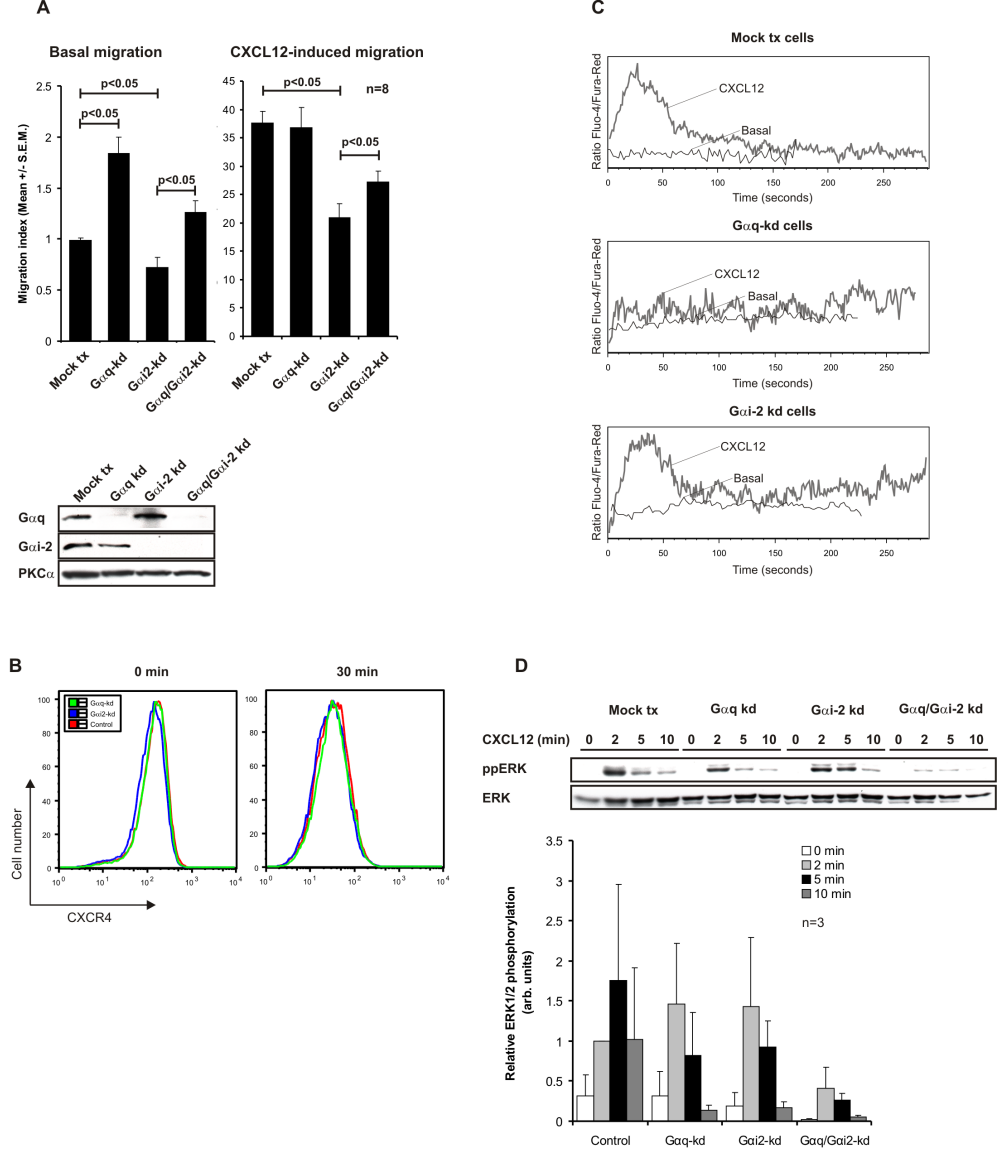
**Gαq and Gαi2 have opposing roles in chemotaxis.** (A) Jurkat TAg T cells were transfected with G_αq_-specific siRNA (GNAQ1103), G_αi2_-specific siRNA (GNAI-2-1050) or control siRNA (GNAQ1103 M3). 48 hours post-transfection cells were placed in Costar Transwell wells with/without 10 ng/ml CXCL12. After 2 hours incubation the numbers of migrated cells were counted by flow cytometry Migration index was calculated as the number of cells migrating divided by the number of basal migrating mock tx cells (n = 8). (B) Cells were subjected to transfection as in (A). 48 hours post-transfection cells were stimulated with CXCL12 for the indicated time points. Cells were stained with anti-CXCR4 and surface expression of the receptor was analysed by flow cytometry (n = 3). (C) Cells were subjected to transfection as in (A). 48 hours post-transfection cells were stained with Fluo-4 and Fura Red AM and calcium fluxes were measured by flow cytometry. Grey lines indicates CXCL12 stimulated cells, black line unstimulated cells. Representative of 3 independent experiments. (D) Cells were subjected to transfection as in (A). 48 hours post-transfection cells were stimulated with CXCL12 for the indicated time points, lysed and subjected to immunoblot analysis with the indicated antibodies. Levels of immunoreactive protein were quantified by densitometric scanning from 3 independent experiments and normalized against total protein (Mean ± S.E.M.).

The reciprocal regulation between the TCR and chemokine receptors [13,22,23] suggest that the response to a specific chemokine is determined both by the differentiation and the activation state of the responding T cell. This implies that migrating T cells are less responsive to activation through the TCR. The physiological relevance of this switch between migration modus and TCR activation modus may be to ensure that migrating T cells do not get distracted by activation cues until they reach their destination. Conversely, TCR triggered T cells are less responsive to chemotactic gradients to ensure optimal activation. We observed that knockdown of G_αq _enhanced basal migration 2-fold (Figure 2A), suggesting that G_αq _may set the threshold for directed migration. This supports the findings of Molon et al. [8] who speculated that CXCR4 signaling through G_αq _promoted adhesion instead of migration. Accordingly, we saw that knockdown of G_αi2 _inhibited directed CXCL12-induced migration, while G_αq _knockdown had no effect (Figure 2A) confirming the notion that G_αi2 _is the principal G_α_-subunit involved in chemotaxis. The inhibitory effect of G_αi2 _knockdown could however be partly reversed with simultaneous knockdown of G_αq _(Figure 2A). We speculate that the presence of G_αq _and absence of G_αi2 _may result in a combined strong inhibitory effect on chemotaxis. Removal of G_αq_, will then lead to partial release of the inhibitory effect of G_αi2 _knockdown alone. Although T cells from G_αq_^-/- ^mice show no migrational defects [7], data from knockout mice are difficult to interpret as compensatory mechanisms may have been established during development. Acute knockdown with siRNA provides an alternative method to examine protein functions. How CXCR4 switches its signaling through G_αi2 _and G_αq _is unknown, but the level of TCR engagement has been implicated [8,13]. Surface expression of CXCR4 following CXCL12-stimulation was neither affected by knockdown of G_αq _nor G_αi2 _during a 1-hour time course (Figure 2B, only 0 minutes and 30 minutes shown). The level of surface expression of CD3 on Jurkat Tags has recently been reported [24]. Furthermore, we suggest that neither Ca^2+^-elevation nor ERK1/2 phosphorylation are involved in chemotaxis as has been suggested in a previous report [7]. While elevation of intracellular Ca^2+ ^was abrogated in the G_αq_-knockdown cells upon CXCR4-stimulation (Figure 2C), it was intact in G_αi2_-knockdown cells. The level of ERK1/2 phosphorylation was similar in both G_αq_-knockdown and G_αi2_-knockdown Jurkat cells. However, concomitant knockdown of both G_αq _and G_αi2 _completely attenuated ERK1/2 phosphorylation (Figure 2D), indicating that this is a G-protein dependent event.

### The G_αi2_-kd-signaling pathway dominates the CXCL12-induced migrational response

**Figure 3:**
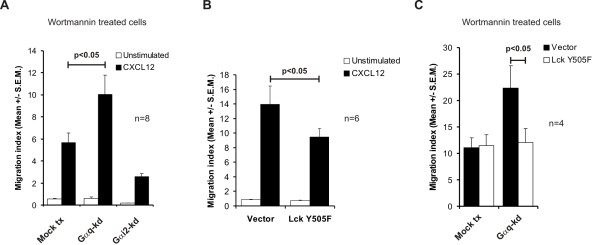
**CXCL12 stimulation primarily activates Gαi2.**(A) Jurkat TAg T cells were tranfected with G-specific siRNA (GNAQ1103), G_αi2_-specific siRNA (GNAI-2-1050) or control siRNA (GNAQ1103 M3). 48 hours post-transfection cells were pre-treated with Wortmannin for 30 minutes and analyzed for CXCL12 dependent and independent migratory responses. Migration index represents fold migration over basal in control cell wells. (B) Jurkat TAg T cells were transfected with Lck Y505F or empty vector migration was analysed in the absence and presence of CXCL12 (10 ng/ml). Migration index represents fold increase in migration over basal migration in wells with control cells. (C) Cells were subjected to tranfection as in (B) pre-treated with Wortmannin as in (A) and migration with CXCL12 was analysed. Migration index represents fold increase in migration over basal migration in wells with control cells.

To test whether the G_αi2_-signaling pathway is the principal pathway activated by CXCR4 we pre-treated cells with the PI3K inhibitor wortmannin prior to incubation with CXCL12. In the presence of wortmannin CXCL12-induced migration was strongly inhibited (Figure 3A vs Figure 2A). Furthermore, in the presence of wortmannin the G_αq_-kd cells displayed an approximately 80% increase in CXCL12-induced migration (Figure 3A). This suggests that the negative regulatory effect of G_αq _on migration is normally not observed when the G_αi2 _signaling pathway through PI3K is intact, and that a switch from G_αi2 _to G_αq _is necessary to fully inhibit migration. These observations correlate well with the data showing that co-knockdown of G_αq _and G_αi2 _has a smaller inhibitory effect on migration than knockdown of G_αi2 _alone (Figure 2A). Although the presence of Lck is shown to be necessary for migration [21] activating Lck through the TCR receptor has an inhibitory effect on migration [13]. Consistent with these findings cells transfected with the constitutively active Lck Y505F showed a significant inhibition of migration compared to vector-transfected cells (Figure 3B). Interestingly, the effect of G_αq_-kd on wortmannin-treated T cells in the presence of CXCL12 is reversed when transfected with Lck Y505F indicating that the effect of G_αq _is mediated through Lck (Figure 3C).

### G_αq_-kd inhibits migration through activation of SHP-1

**Figure 4:**
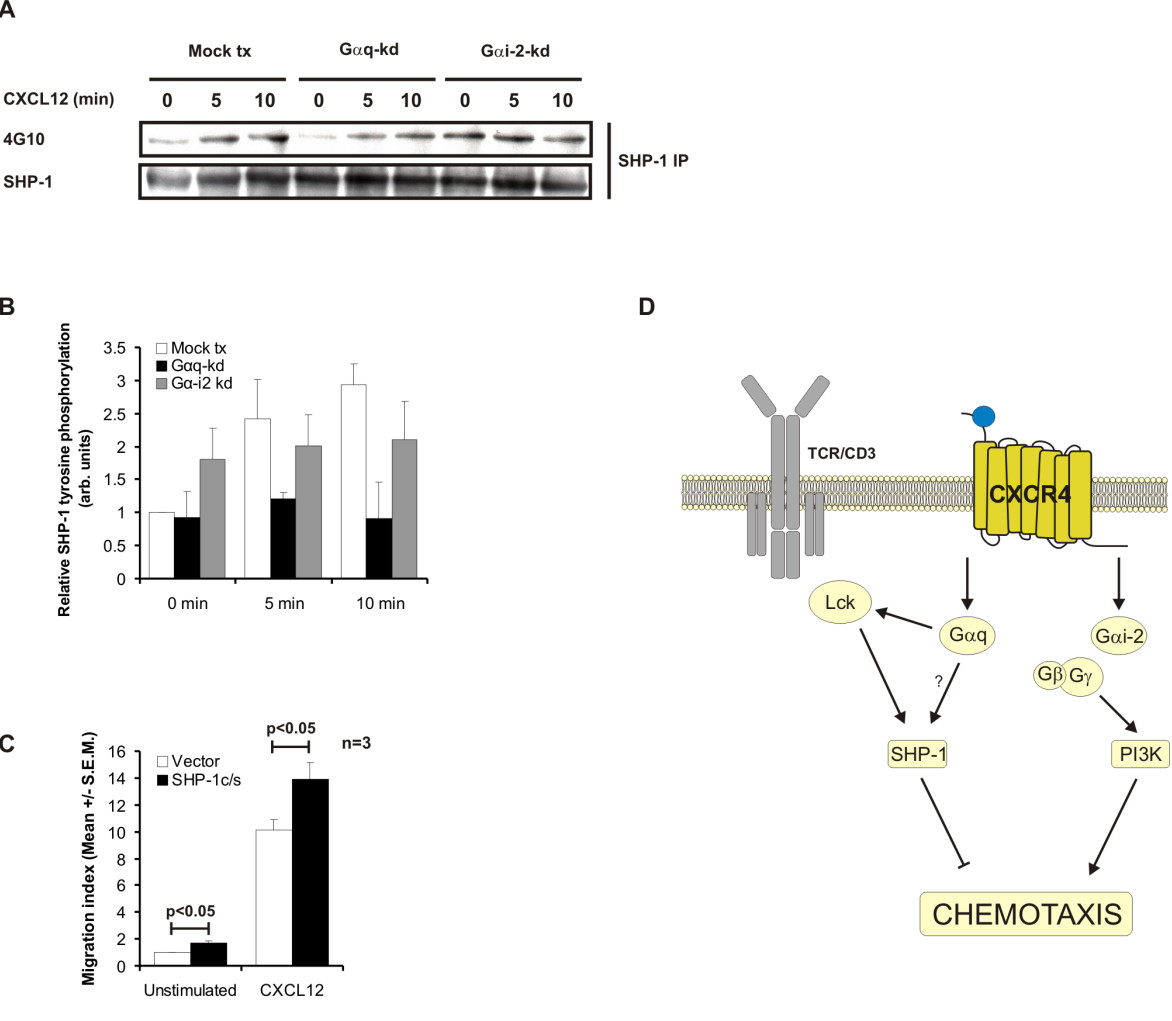
**Migration is inhibited by activation of SHP-1.** (A) Jurkat TAg T cells were transfected with G_αq_-specific siRNA (GNAQ1103), G_αi2_-specific siRNA (GNAI-2-1050) or control siRNA (GNAQ1103 M3). 48 hours post-transfection cells were stimulated with CXCL12 for the indicated time periods and lysed in lysis buffer. SHP-1 immunoprecipitation was performed and immune complexes analysed by immunobloting with the indicated antibodies (n = 3). (B) Immunoblots from 3 independent experiments like in (A) were quantified by densiometric analysis. The graph show mean ± S.E.M. (C) Jurkat TAg T cells were tranfected with the phosphatase defective SHP-1 c/s (C453S) mutant or empty vector. 24 hours post-transfection cells were placed in Costar Transwell plates with or without CXCL12 (10 ng/ml) and migration analysed as above. Migration index represents fold increase in migration over basal migration in wells with vector tx cells (n = 3). (D) Activation of G_αi2 _induces migration through a PI3K pathway whereas activation of G_αq _inhibits migration through an Lck-SHP-1 pathway priming cell for activation through the TCR.

Src homology phosphatase-1 (SHP-1) is an Lck-activated tyrosine phosphatase reported to negatively regulate CXCL12-induced migration [25]. As we have recently reported that G_αq _may activate Lck we examined how G_αq _manipulation would affect SHP-1 regulation in migration. Knockdown of G_αq _resulted in decreased tyrosine phosphorylation of SHP-1 compared to control cells (Fig 4A and 4B). This may indicate a role for G_αq _in the activation of SHP-1. Interestingly, increased basal phosphorylation of SHP-1 was observed in G_αi2 _knockdown cells. This may be due to skewing of the CXCR4 signaling pathway towards G_αq _as there is less G_αi2 _available. Conversely, knockdown of G_αq _might skew the CXCR4 signaling pathway towards G_αi2_. To assess the role of SHP-1 in chemotaxis we transfected Jurkat T cells with the phosphatase-deficient, mutant SHP-1 C453S (c/s) that competes with endogenous SHP-1. Cells transfected with the mutant showed significantly increased basal and CXCL12 induced migration compared to control cells indicating that an Lck-G_αq_-SHP-1 pathway may regulate migration (Fig 4C). Migration is probably not exclusively regulated by SHP-1 and other negative regulators of migration activated by G_αq_-kd might exist.

## Conclusion

We have previously shown that knockdown of G_αq _attenuates TCR-induced Lck activation by an unknown mechanism [16]. While G_αi2 _has a positive role in inducing migration, G_αq _may have a negative regulatory role by promoting Lck activity resulting in increased SHP-1 activity (Figure 4D). Previous reports have demonstrated a reciprocal regulation between the TCR and chemokine-receptors [13,22,23]. Based on our recent report, an optimal TCR-triggering may require the G_αq _signaling pathway as G_αq _knockdown attenuates TCR-induced Lck activation [16]. In contrast, G_αq _knockdown appears to release migration from tonic inhibition. This is in line with data showing increased proliferation and cytokine-production when chemokine-receptors signal through G_αq _[8] suggesting that a switch from G_αi2 _to G_αq _through the chemokine-receptor promotes T cell activation rather than migration. In conclusion, we suggest that G_αq _activation strengthens the commitment of T cells to activation through the TCR while G_αi2 _activation commits the T cell to migration. This might contribute to ensure that T cells migrating towards a site of infection do not get distracted by unnecessary activation cues, while activated T cells are fully committed towards differentiation and proliferation.

## Authors' contributions

All experiments were designed by JN, MI and KT, and conducted by JI and MI. TB contributed with data analysis and co-wrote paper. All authors read and approved the final manuscript.
